# State-Aware Resource Allocation for V2X Communications

**DOI:** 10.3390/s26010344

**Published:** 2026-01-05

**Authors:** Ming Sun, Jinqing Xu, Jiaying Wang

**Affiliations:** 1 College of Computer and Control Engineering, Qiqihar University, Qiqihar 161006, China; 2024911308@qqhru.edu.cn (J.X.); 2024935304@qqhru.edu.cn (J.W.); 2 Heilongjiang Key Laboratory of Big Data Network Security Detection and Analysis, Qiqihar University, Qiqihar 161006, China

**Keywords:** C-V2X, deep reinforcement learning, self-attention, resource allocation

## Abstract

Vehicle-to-Everything (V2X) has become a key technology for addressing intelligent transportation challenges. Improving spectrum utilization and mitigating multi-user interference among V2X links are currently the primary focuses of research efforts. However, the time-varying nature of channel resources and the dynamic vehicular environment pose significant challenges to achieving high spectral efficiency and low interference. Numerous studies have demonstrated the effectiveness of deep reinforcement learning (DRL) in distributed resource allocation for vehicular networks. Nevertheless, in conventional distributed DRL frameworks, the independence of agent decisions often weakens cooperation among agents, thereby limiting the overall performance potential of the algorithms. To address this limitation, this paper proposes a state-aware communication resource allocation algorithm for vehicular networks. The proposed approach enhances the representation capability of observable data by expanding the state space, thus improving the utilization of available observations. Additionally, a conditional attention mechanism is introduced to strengthen the model’s perception of environmental dynamics. These innovative improvements significantly enhance each agent’s awareness of the environment and promote effective collaboration among agents. Simulation results verify that the proposed algorithm effectively improves agents’ environmental perception and inter-agent cooperation, leading to superior performance in complex and dynamic V2X scenarios.

## 1. Introduction

In recent years, the rapidly growing number of private vehicles [1,2] has led to a steady increase in road congestion and traffic accidents [3]. Intelligent Transportation Systems (ITS) [4] and Cooperative Intelligent Transportation Systems (C-ITS) [5] have thus attracted considerable attention from researchers as promising solutions to these challenges. C-ITS facilitates coordinated cooperation among vehicles, infrastructure, and other road users through wireless communication, data exchange, and real-time information sharing. It enhances road safety and traffic flow efficiency while mitigating congestion [6]. Cellular Vehicle-to-Everything (C-V2X) has received extensive attention and rapid development since its inception, owing to its high data transmission rates, low latency, and high reliability [7]. These attributes have established C-V2X as the primary enabling technology for C-ITS. However, due to the highly dynamic nature of vehicular environments—characterized by continuous high-speed mobility—C-V2X must operate under a complex, time-varying, and highly stochastic channel condition when performing resource allocation. This necessitates a high-performance channel resource allocation algorithm to efficiently manage communication resources.

Early research on communication resource allocation algorithms established the fundamental understanding that resource allocation problems in communication systems can be equivalently modeled as optimization problems [8]. Building upon this foundation, the study of communication resource allocation algorithms has roughly undergone several phases: convex optimization and fairness modeling, approximate solution of non-convex problems, distributed game theory and structured approaches, and learning-driven and transferable intelligence. Among these, the convex optimization and fairness phase primarily utilized Lagrangian duality theory and distributed pricing mechanisms to model and solve resource allocation problems, achieving a critical transition from idealized theoretical models to implementable distributed algorithms. This phase constitutes one of the key theoretical frameworks for modern communication resource allocation [9]. Subsequently, to address the widespread non-convex characteristics in practical wireless systems, researchers proposed iterative approximation methods such as sequential convex approximation (SCA) and block successive upper-bound minimization (BSUM), which ensured convergence while maintaining engineering feasibility, and provided “expert algorithms” for subsequent learning-based resource allocation methods to imitate [10]. Furthermore, with the increase in network scale and system autonomy, distributed game theory and structured approaches (such as auction mechanisms and matching theory) were introduced into communication resource allocation research, offering rigorous mathematical tools for resource coordination in multi-agent autonomous systems and becoming an important theoretical framework [11].

Multi-Agent Deep Reinforcement Learning (MADRL) offers the combined advantages of Deep Reinforcement Learning (DRL) and multi-agent collaboration [12]. MADRL aims to enable multiple agents to cooperatively interact within a shared environment to achieve optimal system-wide performance [13]. The inherent capability of MADRL to support cooperative interactions among multiple agents renders it particularly well-suited for addressing resource allocation challenges in distributed vehicular networks. Specifically, MADRL enables vehicle-to-vehicle (V2V) links to collaboratively allocate communication resources, thereby improving the overall V2V transmission success rate and enhancing the throughput of vehicle-to-infrastructure (V2I) links.

Current research primarily focuses on mitigating multi-user interference among V2X links and improving spectrum utilization. Although Multi-Agent Deep Reinforcement Learning (MADRL) has been widely applied to V2X resource allocation [14,15,16,17,18,19,20,21,22,23,24,25] and achieved significant progress, these studies have not explicitly addressed how to enhance cooperation among agents in such environments. In existing approaches, inter-agent collaboration emerges implicitly through individual interactions with the environment. However, since each agent can only observe partial, localized information, the resulting level of cooperation remains limited. The dynamic nature of vehicular networks, combined with the independent decision-making of agents, makes it particularly challenging to improve collaborative capabilities. In V2X resource allocation tasks, poor cooperation often leads multiple links to concentrate on a small subset of channels, significantly increasing mutual interference and causing inefficient resource usage—ultimately degrading system-wide performance.

This paper focuses on addressing three key limitations in current research: limited utilization of observable data by agents, weak environmental perception, and insufficient inter-agent collaboration. Ji et al. [26] took both the agent’s own state information and the state information of surrounding nodes as inputs to a graph neural network GNN. By leveraging the GNN to aggregate the state information of these nodes, they used the aggregated information as the input to a DDQN. Zhang et al. [27], while using a mean-field approach to maintain environmental stationarity, enhanced the representational capacity of the input state with respect to the environment. Both studies improve the environmental representational capability of the input state by augmenting the input information of the reinforcement learning algorithm. Inspired by these studies, this paper innovatively incorporates the historical impact of agents on the environment into the state space and proposes a state-aware resource allocation scheme for vehicular networks. By explicitly modeling agent-environment interactions and enhancing state awareness through conditional attention, the proposed algorithm significantly improves each agent’s perception of the global context. This enhanced awareness enables agents to make more informed and coordinated decisions, thereby fostering effective collaboration even under partial observability and high environmental dynamics. As a result, the proposed approach achieves more balanced channel utilization, reduced interference, and improved overall spectral efficiency in complex V2X scenarios.

The main contributions of this paper are as follows:

This study explicitly embeds the historical impact of agents on the environment into the state space. This innovation enhances the utilization efficiency of observable information and strengthens the representational capacity of inputs for environmental dynamics, thereby providing a crucial foundation for improving the perceptual capabilities of agents.

This paper proposes a conditional attention model, which injects the influence of other agents on the environment as conditional information into the backbone network. This significantly enhances the model’s perception of the environment, enabling it to sensitively capture environmental changes and thereby improve cooperation among different agents.

**Figure 1:**
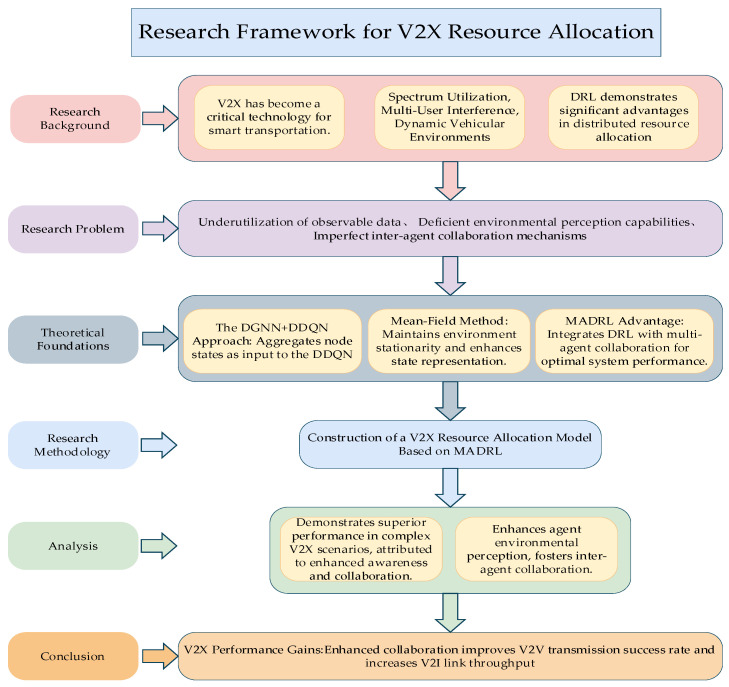
Research Framework for V2X Resource Allocation.

The research framework diagram of this paper is shown in Figure 1. The remainder of this paper is organized as follows: Section 2 reviews related work. Section 3 introduces the C-V2X communication network model used in this paper, the architecture of the agents, the information aggregation method, and the state-aware network design. Section 4 presents the simulation results.

## 2. Related Work

In the early stages of research on vehicular network resource allocation, researchers primarily focused on traditional methods to address these challenges. Sun et al. [28] proposed the Separate resource block and power allocation (SOLEN) algorithm, which first transforms the problem into a maximum weighted matching (MWM) problem on a bipartite graph and solves it efficiently using the Hungarian algorithm, ensuring orthogonal resource allocation and initial satisfaction of power constraints. Subsequently, based on the allocated resource blocks, power is further optimized via convex optimization and dual decomposition to improve cellular user rates while strictly meeting the signal-to-interference-plus-noise ratio (SINR) requirements of V2V links. Mei et al. [29] introduced a Long-Term Evolution (LTE)-based resource allocation scheme for V2V communication, aiming to jointly optimize wireless resources, power allocation, and modulation and coding schemes (MCS) to meet specific latency and reliability requirements. Ashraf et al. [30] proposed a decentralized algorithm to jointly optimize transmission delay and success probability. Yang et al. [31] formulated a dual-timescale resource allocation framework, addressing both long-term mobility patterns and short-term channel variations. Wang et al. [32] designed a hybrid architecture combining decentralized clustering, inter-cluster distributed decision-making, and intra-cluster centralized communication to balance scalability and performance. In these conventional approaches, resource allocation is typically formulated as an optimization problem-often NP-hard-making it computationally challenging to solve, especially in large-scale or dynamic environments. Moreover, traditional optimization methods exhibit significant limitations when dealing with complex, uncertain, or rapidly changing conditions. Additionally, heuristic-based resource allocation algorithms generally require a multi-step process involving information collection, centralized computation, and command dissemination. This procedure introduces substantial time overhead, making such approaches less suitable for highly dynamic vehicular networks where low-latency responses are critical.

Following the introduction of the Deep Q-Network (DQN) algorithm [33], researchers began to adopt deep reinforcement learning (DRL)-based approaches for solving resource allocation problems in vehicular networks. In [14], each V2V link is modeled as an independent agent, with agents making decentralized decisions during execution. The framework adopts the centralized training with decentralized execution (CTDE) paradigm and introduces low-dimensional action–observation histories—referred to as fingerprints—to address the non-stationarity issue inherent in multi-agent environments. Compared to random and several traditional baseline methods, multi-agent reinforcement learning (MARL) algorithm significantly improves the sum throughput of V2I links and enhances the success rate of V2V messages delivered within their latency budgets. Although this study has certain limitations-such as limited inter-agent collaboration and relatively low V2V link throughput-it represents one of the early pioneering efforts to apply reinforcement learning to resource allocation in vehicular networks, thus greatly stimulating subsequent research in this field. Building upon the foundation laid by [14], researchers have subsequently introduced more advanced DRL algorithms into vehicular communication resource allocation, including Double DQN (DDQN) [34] and Dueling Double DQN (D3QN) [35], aiming to further improve training stability, action evaluation accuracy, and overall system performance.

Reference [18] proposes a two-stage, dual-model resource allocation algorithm that employs the DQN algorithm for channel assignment and the DDPG algorithm for power control. This approach ensures reliable transmission performance while achieving high robustness and low bit error rate. Reference [19] designs a centralized vehicular communication resource allocation algorithm using the DQN framework. The algorithm treats channel allocation and power control as a joint optimization problem and leverages reinforcement learning to maximize spectrum utilization while minimizing multi-user interference among V2X links. In [20], the D3QN framework is applied to vehicular communication resource allocation. The study demonstrates the effectiveness of reinforcement learning in addressing resource allocation under spectrum-constrained scenarios, showing superior performance in dense and dynamic network environments. Reference [21] proposes a communication mode selection model based on the DQN algorithm, which enables V2V links to dynamically select appropriate communication modes (e.g., direct V2V or infrastructure-assisted) according to the observed state. This adaptive selection mechanism achieves high reliability and low communication latency, enhancing the overall efficiency and resilience of the network.

Although these studies [18,19,20] have successfully applied deep reinforcement learning algorithms to vehicular communication resource allocation and achieved promising performance—demonstrating the effectiveness of such approaches in this domain and promoting further research progress-they remain primarily exploratory in nature. These works do not incorporate environment-specific adaptations tailored to the highly dynamic and time-varying characteristics of vehicular networks. In particular, they fail to address two critical challenges: the limited environmental perception capability of individual agents and the insufficient level of cooperation among multiple agents in complex V2X scenarios. As a result, there remains a need for more advanced frameworks that enhance situational awareness and foster collaborative decision-making to fully unlock the potential of multi-agent learning in realistic vehicular environments.

Reference [15] investigates the challenges of limited observability for individual agents and unstable joint training in the context of vehicular communication resource allocation. In this framework, each V2V link is modeled as an independent agent, and D3QN is employed within each agent to reduce action-value estimation bias and improve learning efficiency. Furthermore, the study integrates Federated Learning (FL) into the multi-agent training process: each agent trains its D3QN model locally using private experience data, while a central server periodically aggregates the local model parameters through federated averaging and broadcasts the updated global model back to the agents. This approach avoids direct transmission of raw data, thereby reducing communication overhead and enhancing privacy protection. Experimental results show that the proposed method outperforms baseline schemes—both with and without FL or D3QN—in terms of cellular network sum rate and V2V packet transmission success rate. Reference [26] addresses the issue of high environmental dynamics in vehicular networks by introducing a Graph Neural Network (GNN) as a feature extractor within the deep reinforcement learning framework. The extracted features are then fed into a DDQN model for decision-making. By incorporating GNNs, the algorithm enhances the representation capability of spatial-temporal network states, significantly improving agents’ perception of their environment and leading to better resource utilization. However, this approach requires collecting information from neighboring nodes for message passing and aggregation, which introduces additional computational and communication latency. Reference [27] tackles the scalability challenge in large-scale, high-density vehicular networks, where traditional Multi-Agent Reinforcement Learning (MARL) becomes infeasible due to the exponential growth in interaction complexity as the number of agents increases. To address this, the study proposes a Mean-Field Multi-Agent Reinforcement Learning (MF-MARL) framework, in which each agent does not interact directly with every other agent. Instead, agents make decisions based on the average effect or mean-field approximation of the entire population’s behavior. This simplification enables scalable learning and coordination in massive vehicular networks, achieving efficient resource allocation without requiring full peer-to-peer observation or communication.

These studies [15,26,27] not only recognize the advantages of deep reinforcement learning in vehicular communication resource allocation but also adapt reinforcement learning frameworks to address the unique characteristics of this domain. While these works have further advanced the application and development of deep reinforcement learning in vehicular networks, they still fall short in fostering effective cooperation among agents. Specifically, the interactions between different agents remain largely independent or implicitly coordinated, without explicit mechanisms to enhance collaborative decision-making. As a result, the potential for synergy among agents is not fully exploited, limiting the overall system performance in highly dynamic and interference-prone environments.

To enhance the utilization of observable information, improve agents’ perception of the environment, and strengthen cooperation among agents, this paper proposes a state-aware communication resource allocation algorithm for vehicular networks. The proposed algorithm incorporates the impact of each agent on the environment as a conditional input. To maximize the utilization of observable information, this environmental impact is extended from the current time step to a historical time window, capturing temporal dynamics and long-term interactions. Furthermore, additional contextual information from the environment-such as channel states, interference levels, and mobility patterns-is incorporated into the state representation. This state information is repeatedly injected into the proposed state-aware backbone network at multiple stages during the learning process. By conditioning the network on both the agent’s influence and rich environmental context throughout the decision-making pipeline, the model achieves significantly enhanced environmental awareness. This improved perception enables agents to better anticipate the consequences of their actions and respond cooperatively to the behaviors of others, thereby promoting effective collaboration in dynamic and complex V2X scenarios.

## 3. System and Model

In the context of resource allocation for vehicular networks, all vehicles operate within a highly dynamic environment characterized by high mobility. This renders it extremely challenging for centralized resource allocation algorithms to timely and accurately acquire the channel state information (CSI) from all vehicles. Consequently, this paper adopts a distributed approach for channel resource allocation. In this section, we first present the system model, followed by a detailed description of the proposed channel resource allocation algorithm.

### 3.1. System Model

**Figure 2:**
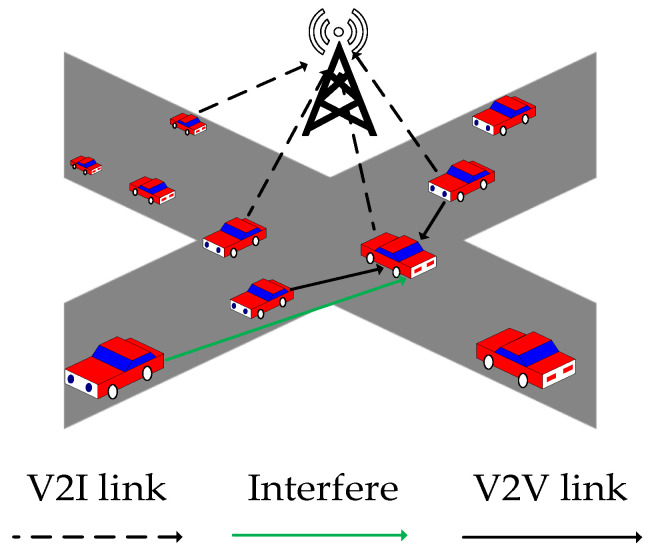
C-V2X System Model.

This paper considers an orthogonal frequency-division multiple access (OFDMA) C-V2X network, which is located in an urban model. As shown in Figure 2, the model consists of N vehicles and a base station located at the center of the C-V2X cellular network, each equipped with a single antenna. There are *N* V2I links and *K* V2V links in the V2X cellular network. V2I communication is implemented using the direct communication mode discussed in [7], while V2V communication is implemented using the PC5 interface in device-to-device (D2D) communication [7,13,36]. The set of V2I links is denoted by $V_{i}=\{1,\ldots ,n,\ldots ,N\}$, the set of V2V links by $V_{v}=\{1,\ldots ,k,\ldots ,K\}$, the set of vehicles by *U* = {1, …, *n*, …, *N*}, and the set of orthogonal subchannels by *C* = {1, …, *l*, …, *L*}. Each vehicle can be equipped with one V2I link and multiple V2V links. In this paper, the set *C* of orthogonal subchannels is evenly allocated to different V2I links, and a fixed transmission power is assigned [37]. The C-V2X network scenario designed in this paper refers to the urban cases in [12].

The channel gain in the C-V2X network is calculated in this paper as follows:

(1) $$g_{u,b}^{i,x}=h_{u,b}^{i,x}\cdot \beta_{u,b}^{i,x}$$

(2) $$g_{u,e}^{v,k}=h_{u,e}^{v,k}\cdot \beta_{u,e}^{v,k}$$

$g_{u,b}^{i,x}$ represents the channel gain of the *x*-th V2I link, where the transmitter is vehicle *u* and the receiver is the base station *b*. Here, u∈U and $x\in V_{i}$ are sets; $g_{u,e}^{v,k}$ represents the channel gain of the *k*-th V2V link, where the transmitter is vehicle *u* and the receiver is vehicle *e*. Here, $e\in U,k\in V_{v}$. $h_{u,b}^{i,x},h_{u,e}^{v,k}$ represents the frequency-dependent small-scale fading effect, which is assumed to follow an exponential distribution. $\beta_{u,b}^{i,x},\beta_{u,e}^{v,k}$ represents the large-scale fading effect, which includes path loss and shadowing, and is assumed to be frequency-independent. The modeling data for small-scale fading and large-scale fading are defined in Table 1.

In the C-V2X cellular network considered in this paper, the SINR of the *x*-th V2I link is denoted by $\gamma_{u,b}^{i,x}$:

(3) $$\gamma_{u,b}^{i,x}=\frac{P_{x}^{i}\cdot g_{u,b}^{i,x}}{\sum_{k\in V_{v}}\rho_{x,k}^{i,v}\cdot P_{k}^{v}\cdot g_{u,b}^{v,k}+\sigma^{2}}$$

where $P_{x}^{i}$ is the transmission power of the *x*-th V2I link, $g_{u,b}^{i,x}$ is the channel gain of the *x*-th V2I link, $\sum_{k\in V_{v}}\rho_{x,k}^{i,v}\cdot P_{k}^{v}\cdot g_{u,b}^{v,k}$ represents the interference from V2V links on the *x*-th V2I link. Where $\rho_{x,k}^{i,v}\in [0,1]$, $\rho_{x,k}^{i,v}=1$ represents that the *k*-th V2V link uses the same channel as the *x*-th V2I link, while $\rho_{x,k}^{i,v}=0$ represents that the *k*-th V2V link uses a different channel from the *x*-th V2I link. $P_{k}^{v}$ is the transmission power of the *k*-th V2V link, $g_{u,b}^{v,k}$ is the channel gain generated by the *k*-th V2V link, and $\sigma^{2}$ is the noise power. The SINR of the *k*-th V2V link is given by:

(4) $$\gamma_{u,e}^{v,k}=\frac{P_{k}^{v}\cdot g_{u,e}^{v,k}}{\sum_{m\in V_{v},m\neq k}\sum_{z\in U}\rho_{k,m}^{v,v}\cdot P_{m}^{v}\cdot g_{z,e}^{v,m}+\sum_{x\in V_{i}}\rho_{k,x}^{v,i}\cdot P_{x}^{i}\cdot g_{u,e}^{i,x}+\sigma^{2}}$$

where $P_{k}^{v}$ is the transmission power of the *k*-th V2V link, and $g_{u,e}^{v,k}$ is the channel gain of the *k*-th V2V link. $\sum_{m\in V_{v},m\neq k}\sum_{z\in U}\rho_{k,m}^{v,v}\cdot P_{m}^{v}\cdot g_{z,e}^{v,m}$ represents the interference from other V2V links on the *k*-th V2V link. $\rho_{k,m}^{v,v}=[0,1]$, $\rho_{k,m}^{v,v}=1$ represents that the *k*-th V2V link uses the same channel as the *m*-th V2V link, while $\rho_{k,m}^{v,v}=0$ indicates that the *k*-th V2V link uses a different channel from the *m*-th V2V link. $P_{m}^{v}$ is the transmission power of the *m*-th V2V link, and $g_{z,e}^{v,m}$ is the channel gain of the *m*-th V2V link. $\sum_{x\in V_{i}}\rho_{k,x}^{v,i}\cdot P_{x}^{i}\cdot g_{u,e}^{i,x}$ represents the interference from the V2I links. $\rho_{k,x}^{v,i}=[0,1]$, $\rho_{k,x}^{v,i}=1$ represents that the *x*-th V2I link uses the same channel as the *k*-th V2V link, while $\rho_{k,x}^{v,i}=0$ represents that the *x*-th V2I link uses a different channel from the *k*-th V2V link. The transmission rate of the *x*-th V2I link is $V_{x}^{i}$, and the transmission rate of the *k*-th V2V link is also $V_{k}^{v}$. The calculation formula for transmission rate is expressed as follows:

(5) $$V_{x}^{i}=\log_{2}\left(1+\gamma_{u,b}^{i,x}\right)$$

(6) $$V_{k}^{v}=\log_{2}\left(1+\gamma_{u,e}^{v,k}\right)$$

For V2V data packet transmission, it is assumed that each V2V transmitter has a data transmission queue. The V2V transmitter periodically generates safety data of size *B* and writes it into the transmission queue. The *k*-th V2V link consumes data from the queue at a rate $V_{k}^{v}$. At time *t*, the remaining data in the queue is given by Equation (7), *t* is reset at the end of each transmission cycle, *t* has an upper bound *T*, which is set to *T* = 100 ms, and *W* is the transmission bandwidth. Therefore, the time required to complete the data transmission is given by Equation (8). The transmission result of the *k*-th V2V link is $P_{k}^{s}$ (Equation (9)), and the global transmission success rate $p^{g}$ is given by Equation (10).

(7) $$Q_{t}=max\left(0,B-\sum_{t=1}^{T}t\cdot W\cdot V_{k}^{v}\right)$$

(8) $$T^{\prime }=\left\{\begin{array}{l} T^{\prime }(t+1),\;\left(Q_{t}\neq 0\right)\\ t,\;\;\;\;\;\;\;\;(Q_{t}=0) \end{array}\right.$$

(9) $$P_{k}^{s}=\left\{\begin{array}{l} 1,\;(Q_{t}=0)\\ 0,\;(Q_{t}\neq 0) \end{array}\right.$$

(10) $$p^{g}=\frac{\sum_{k\in K}p_{k}^{s}}{K}\;$$

### 3.2. Problem Definition

The objective of the proposed algorithm is to design an efficient channel resource allocation scheme for V2V links, aiming to maximize the transmission success rate of V2V links, maximize the communication rate of V2I links, and minimize the transmission delay of V2V data packets.

(11) $$\;\underset{\rho_{x,k}^{i,v},\rho_{m,k}^{v,v}}{\max}\frac{1}{T}\frac{1}{N}\sum_{t=1}^{T}\sum_{x=1}^{N}V_{x}^{i}\;$$

(12) $$\underset{\rho_{x,k}^{i,v},\rho_{m,k}^{v,v}}{\max}\frac{\sum_{k\in V_{v}}p_{k}^{s}}{K}\;$$

(13) $${\underset{\rho_{x,k}^{i,v},\rho_{m,k}^{v,v}}{\min}}^{T^{\prime }}=\left\{\begin{array}{l} T^{\prime }(t+1),\;\left(Q_{t}\neq 0\right)\\ t,\;\;\;\;\;\;\;\;(Q_{t}=0) \end{array}\right.$$

(14) $$x\in V_{i},k\in V_{v},\rho_{x,k}^{i,v}\in \{0,1\},\rho_{m,k}^{v,v}\in \{0,1\}$$

By examining Equations (11)–(14), it can be observed that the problem is a multi-objective optimization task, whose computational complexity increases significantly with the number of V2V links and the number of communication resources. To address this challenge, this paper employs a MARL framework. In this work, we adopt a CTDE paradigm, where agents are trained using global information but operate based only on local observations during execution.

### 3.3. A State-Aware Communication Resource Allocation Algorithm for Vehicular Networks

#### 3.3.1. State and State Space

In the proposed algorithm, each agent can only observe its own local information and has no access to the information of other agents. The state observed by the *m*-th agent at time *t* is denoted by $S_{t,m}$.

(15) $$S_{t,m}=\left\{G_{t,m},I_{t,m},F^{\prime },F^{m},ƛ\right\}$$

$G_{t,m}=\left\{g_{m},g_{m}^{\prime },g_{B}\right\}$ denote the set of channel gains. $g_{m}$ represents the state of the channel allocated to agent *m*, $g_{m}^{\prime }$ denotes the gains received by agent *m* on other channels, and $g_{B}$ denotes the gains from its own transmitter to the base station. $I_{t,m}=\left\{i_{1},\ldots ,i_{c},\ldots ,i_{C}\right\}$ denote the set of interference, and let $i_{c}$ represent the interference on the *c*-th channel, which can be measured by the receiver of agent *m* [29,38]. $F^{\prime }=\left\{s,epo\right\}$ is a low-dimensional fingerprint set, which can effectively mitigate the non-stationarity problem arising from multi-agent interactions [14,15], *s* denotes the stochastic exploration rate, and *epo* represents the current training epoch. ƛ denotes the ratio of the remaining transmission payload to the current transmission load. $F^{m}=\left\{f_{1}^{m},\ldots ,f_{l}^{m},\ldots ,f_{L}^{m}\right\}$ denotes the aggregated transmission rate of agent *m* over each action within the δ time slots, initialized to zero.

#### 3.3.2. Action Space

The proposed resource allocation algorithm assigns a spectrum resource to each link. Therefore, the action space is defined as the set of channels C, and the dimensionality of the action space is equal to the number of orthogonal channels *L*.

#### 3.3.3. Reward Function

In reinforcement learning, the reward function is of paramount importance as it determines the evolutionary direction of the agents. It can be readily observed from Equation (7) that a high sum of transmission rates contributes to achieving the objective in Equation (7). Therefore, the reward function in this paper is defined as:

(16) $$\;r=\alpha \cdot \frac{1}{n}\sum_{j=1}^{n}V_{j}^{i}+\beta \cdot \frac{1}{k}\sum_{j=1}^{k}V_{j}^{v}$$

where $\sum_{j=1}^{n}V_{j}^{i}$ denotes the sum of transmission rates of all V2I links, $\sum_{j=1}^{k}V_{j}^{v}\;$ denotes the sum of transmission rates of all V2V links, and α,β are scaling factors.

#### 3.3.4. Network Architecture

**Figure 3:**
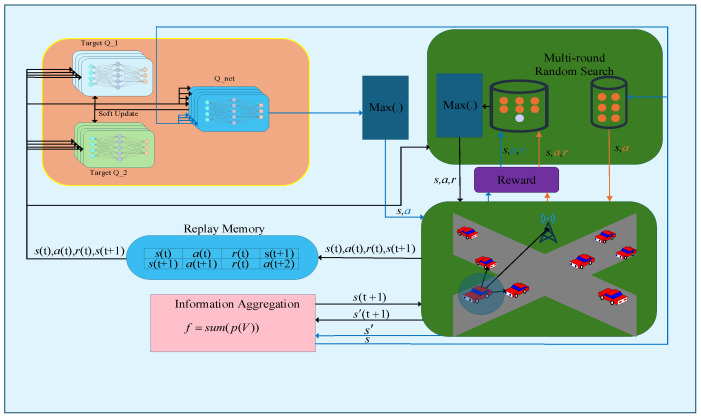
Intelligent Architecture.

Figure 3 illustrates the overall network architecture of the agent, which comprises three Q-networks. To mitigate the maximization bias problem, a multi Q-network design is adopted in this paper, minimizing the impact of maximization bias by taking the minimum value among the critics. In reinforcement learning algorithms, the update of the value network typically employs temporal difference methods, a process that can easily lead to value overestimation bias and the cumulative error issues associated with “bootstrapping” [30]. To mitigate these problems, researchers have proposed two key improvements: first, separating the action selection network from the action evaluation network [30]; and second, using multiple value networks and selecting the minimum value among them for updates [39,40]. These methods have become common training techniques in modern deep reinforcement learning. This paper adopts a similar design, employing an architecture where the action selection and evaluation networks are separated during value network training, and utilizing multiple value networks with a minimum value selection approach to suppress bias. The number of action evaluation networks influences both algorithm performance and the total number of model parameters. Following the approach in [40], this paper uses two independent action evaluation networks. The Replay Memory module collects information obtained from the environment and stores each experience as a training sample in the form of (*s*(*t*), *a*(*t*), *r*(*t*), *s*(*t* + 1)), which is subsequently fed into the network for training. The C-V2X network implemented in this study realistically simulates vehicles, the surrounding environment, and their corresponding channel models. Based on the current channel state, each agent selects the communication channel. Within the simulation, the channel fading information is updated every 1 ms, while the vehicle positions are updated every 1 s. After the Q-net receives the state information, it generates an action, while Multi-round Random Search simultaneously produces multiple candidate actions. These actions are applied to the environment, and the one yielding the highest reward is selected and stored in the Replay Memory. The Q-net is then trained by sampling multiple transitions from the Replay Memory. Multi-round Random Search [36] is a strategy designed to accelerate network convergence by performing multiple rounds of random exploration, thereby enhancing the agent’s ability to explore the environment effectively and discover high-reward policies in complex and dynamic scenarios.

Each vehicle can communicate with multiple neighboring vehicles; therefore, the number of communication links associated with each vehicle exceeds two (one V2V and one V2I link). In this paper, the transmission rates of all V2V links on a given vehicle are aggregated and used as conditional information input to the model. We propose an innovative information aggregation algorithm that leverages transmission rates as effective indicators of channel conditions. The communication rate of a link reflects the underlying channel state, and over a reasonable time period, the accumulated transmission rate exhibits strong representational power for characterizing channel quality. Therefore, the proposed information aggregation algorithm processes the transmission rates over δ consecutive time slots to capture temporal channel dynamics and enhance decision-making performance. First, a transmission rate vector initialized to zero is assigned to each V2V link. Each such vector has L elements, where L denotes the size of the action space, and contains only one non-zero element $f_{l}^{k}$ at the position corresponding to the selected action. The proposed information aggregation algorithm is described as follows:

(17) $$F_{t}^{k}=\left\{\begin{array}{l} V_{k}^{v},V_{k}^{v}>\tau \\ \sigma ,V_{k}^{v}<=\tau \end{array}\right.$$

(18) $$f_{l}^{k}=\sum_{t=1}^{\delta }F_{t}^{k}\cdot I_{l}^{t}$$

$V_{k}^{v}$ denotes the output rate of the *k*-th V2V link, and $F_{t}^{k}$ is an intermediate variable used in the information aggregation process. $I_{l}^{t}\in \{0,1\}$ is an indicator: $I_{l}^{t}=1$ if the *k*-th V2V link uses channel *l*, and $I_{l}^{t}=0$ otherwise.

**Figure 4:**
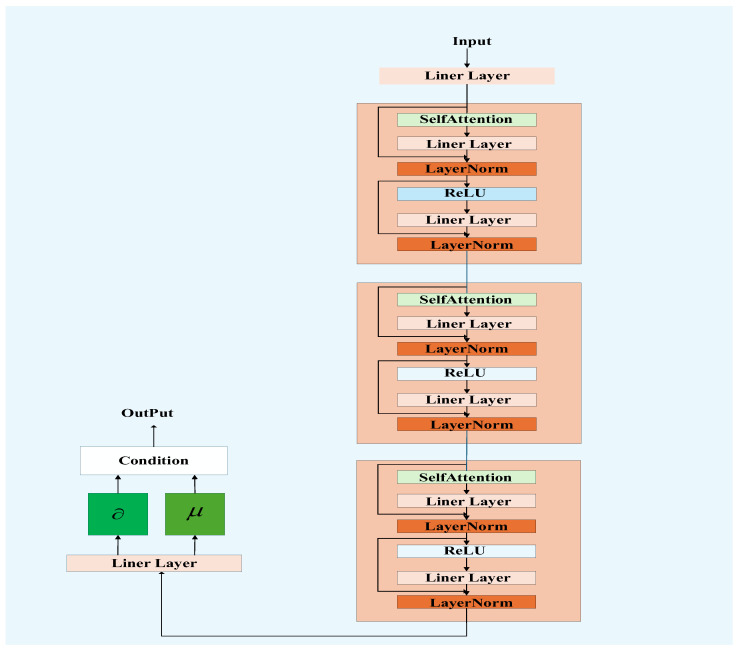
Network Architecture.

In this paper, all Q-networks share the same network architecture, as illustrated in Figure 4. After each agent feeds its locally observed state information into the model, the input first undergoes an embedding layer, followed by three identical attention modules in sequence. The output is then passed to a prediction head, which generates an *L*-dimensional scaling vector and an *L*-dimensional rotation vector. These two vectors are subsequently used to scale and rotate the *L*-dimensional conditional information, respectively, enabling adaptive feature modulation based on the current state.

The proposed “conditional attention” mechanism in this study fundamentally operates by dynamically generating condition-aware feature transformation parameters through a network composed of multiple attention modules. Specifically, the mechanism takes state S, containing specific conditional information F as input. This network consists of several serially connected attention modules with identical structures, each comprising a self-attention layer, linear layers, normalization layers, and activation functions. Within each module, dual residual connections ensure the preservation and flow of conditional information F throughout the computation. Through the cascading of multiple modules and residual connections, the conditional information permeates the entire forward propagation process. The final output of the network is a set of rotation matrices and scaling matrices, which are then applied to the original features to perform a conditional rotation and scaling operation.

Traditional feedforward layers are inherently static, relying on fixed-weight affine transformations and pointwise nonlinear activations, resulting in a consistent feature processing pattern across all input samples. In contrast, the conditional attention mechanism employs self-attention operations to dynamically recalibrate weights based on the relationships among elements within the current input sequence. This enables the model to adaptively focus on the subset of features most relevant to the current conditions, thereby achieving a more refined and context-sensitive nonlinear representation of environmental states.

The training procedure of the proposed channel resource allocation algorithm is presented in Algorithm 1.
**Algorithm 1.** Proposed Algorithm1: Initialize net $Q\_\mathrm{net}(\cdot ;\theta ),\mathrm{target}\;Q\_1(\cdot ;\theta_{1}),\mathrm{target}\;Q\_2(\cdot ;\theta_{2})$ with the weights 2: for: episode = 1, …I: 3:     for: t = 1, …*T*: 4:      for: k = 1, …*N*: 5:         Observation state s 6:         Information Aggregation 7:         Select action at according to Q net. 8:         Execute the random search algorithm to optimize the actions. 9:         Obtain the reward r and the next state s(t + 1). 10:       Randomly sample a mini-batch (s, a, r, s’) from -Experience Replay. 11:       Update $Q\_\mathrm{net}(\cdot ;\theta ),\mathrm{target}\;Q\_1(\cdot ;\theta_{1}),\mathrm{target}\;Q\_2(\cdot ;\theta_{2})$ 12: end

## 4. Simulation Results

In this section, the proposed resource allocation algorithm is evaluated through simulation experiments. The simulation is implemented in Python using PyTorch (version 2.3.0) to construct the network architecture. The experiments are conducted on a PC equipped with a CPU (32 GB RAM) and a GPU (NVIDIA RTX 4070 Super, 12 GB VRAM). The simulation environment is configured strictly according to the urban scenario defined in 3GPP TR 36.885 [27], Annex A, including parameters such as the number of lanes, transmit power levels, bandwidth, and vehicle mobility speed. The key simulation parameters are listed in Table 1 and Table 2, and unless otherwise specified, all default experimental settings are based on these tables. Each agent consists of three Q-networks, and is trained for 11,000 iterations. The Q-network employs the Mean Squared Error (MSE) loss function and is optimized using the Adam optimizer with a learning rate of 0.00001. The batch size is set to 512, and the experience replay buffer has a capacity of 1024, with stored transitions updated dynamically over time. To ensure statistical reliability, performance metrics reported in the simulations are averaged over 4000 interactions with the environment, except for quantities that cannot be meaningfully averaged (cases where averaging is not applied are explicitly noted in the text). The evaluation metrics include: the average transmission rate of V2I links, the throughput per transmission period of V2V links, and the communication delay of V2V links. This section first presents training-related results of the proposed algorithm, including the reward curve and ablation studies. Subsequently, comparative simulation results are provided to demonstrate the performance of the proposed algorithm across the aforementioned evaluation metrics.

**Table 1:** V2X Network Parameters Parameter.

	V2I Link	V2V Link
Path loss model	128.1 + 37.6log10 d, d in km	LOS in WINNER +B1 Manhattan
Shadowing distribution	Log-normal	Log-normal
Shadowing standard deviation ξ	8 dB	3 dB
Decorrelation distance	50 m	10 m
Path loss and shadowing update	A.1.4 in every 100 ms	A.1.4 in every 100 ms
Fast fading	Rayleigh fading	Rayleigh fading
Fast fading update	Every 1 ms	Every 1 ms

**Table 2:** Channel Model Parameters.

Parameter	Value
Carrier frequency	2 GHz
Bandwidth	1.5 MHz
BS antenna height	25 m
BS antenna gain	8 dBi
BS receiver noise figure	5 dB
Vehicle antenna height	1.5 m
Vehicle antenna gain	3 dBi
Vehicle receiver noise figure	9 dB
Absolute vehicle speed v	From 36 to 54 km/h
Vehicle drop and mobility model	Urban case of A.1.2
V2I transmit power $p^{c}$	23 dBm
Noise power $\sigma^{2}$	−114 dBm
Time constraint of V2V payload transmission T	100 ms
V2V payload size B	[1, 2, …] × 1060 Bytes
V2V transmit power $p^{d}$	23 dBm

To better evaluate the proposed algorithm, this paper introduces the following baseline and comparative algorithms:

DDQN-CA: The proposed state-aware communication resource allocation algorithm based on DDQN.

DDQN: The DQN-based resource allocation algorithm proposed by Liang [20], but with the network implemented using only an MLP, serving as a baseline for DDQN.

DDQN-C: The proposed state-aware communication resource allocation algorithm, but with the network implemented using only an MLP.

DDQN-A: The proposed state-aware communication resource allocation algorithm without the use of information aggregation.

GNN: An algorithm proposed by Ji [26] that combines GNN with reinforcement learning. This algorithm exhibits strong state perception capabilities but requires the collection of information from neighboring nodes. Graph Neural Networks and Deep Reinforcement Learning-Based Resource Allocation for V2X Communications.

GA: GA is a well-established heuristic optimization method that has demonstrated strong performance in solving various complex optimization problems. However, due to its lack of adaptability and dynamic decision-making capability, GA requires multiple iterations over the joint action space at each time step, along with continuous feedback from the environment. This high computational overhead and latency make the genetic algorithm difficult to apply in real-world V2X networks, where timely and responsive resource allocation is critical.

In DRL-based methods, the process of updating network parameters typically plays a dominant role. Consider a DRL agent with H hidden layers, where the h-th hidden layer (*h* = 1, 2, …, *H*) contains h neurons. Let *Z* denote the dimension of the input layer, and *O* denote the dimension of the output layer (generally corresponding to the size of the action space). The number of trainable parameters in such a network architecture is $X=3(Zx_{1}+\sum_{h=1}^{H}x_{h}x_{h+1}+o)$, with a computational complexity of O(BX), where *B* is the batch size. The embedding dimension *d* is 128.

DDQN, DDQN-C, GNN: The neural networks used in DDQN, DDQN-C, and GNN all adopt a three-layer fully connected architecture consistent with neuron counts of 520, 250, and 120 per layer, respectively. The GNN algorithm consists of a centralized graph neural network and a DDQN-based reinforcement learning algorithm. The trainable parameters of the graph neural network component are significantly fewer than those of the DDQN component, making the total trainable parameters of the GNN algorithm approximately equal to O(BX). Consequently, the computational complexities of DDQN, DDQN-C, and GNN are O(BX), O(BX+do), and O(BX), respectively.

DDQN-A, DDQN-CA: The DDQN algorithms based on the attention mechanism consist of 3 attention modules and an output head. The embedding dimension for each vector is *d* = 128, and the trainable parameters for each attention module are $O(6d^{2}+10d)$. DDQN-CA includes one additional output head compared to DDQN-A. Therefore, the computational complexities of DDQN-A and DDQN-CA are O(ℑBX+do) and O(ℑBX+2do), respectively, where ℑ≈1.6.

Based on the computational complexity analysis, the DDQN algorithm exhibits the lowest complexity but also the poorest performance. In contrast, while the DDQN-CA algorithm has the highest number of trainable parameters, it achieves the best performance. Comparatively, the GNN algorithm appears to strike a favorable balance between performance and complexity. However, it relies on a centralized processing mechanism, which requires aggregating information from surrounding nodes via a graph neural network as input for the current node, thereby introducing additional communication overhead and time latency. Therefore, despite the trainable parameters of the DDQN-CA algorithm increasing by approximately ℑ times, the significant performance improvement it delivers remains acceptable.

**Figure 5:**
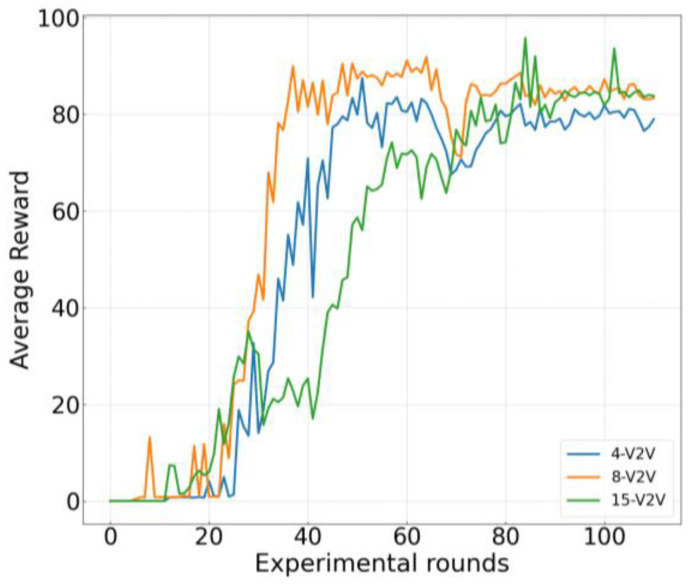
Training Reward.

Figure 5 illustrates the variation in the training reward with respect to the number of training iterations under three different parameter settings in the proposed algorithm. The setting labeled “4-V2V link” corresponds to a scenario with 4 V2V links and 4 V2I links, where the packet size to be transmitted over each V2V link is 5 × 1060 Bytes. The “8-V2V link” setting includes 8 V2V links and 4 V2I links, with a packet size of 3 × 1060 Bytes per V2V link. The “15-V2V link” setting comprises 15 V2V links and 15 V2I links, also with a packet size of 3 × 1060 Bytes per V2V link. As shown in the figure, the reward function increases with the number of iterations across all three configurations, indicating that the network is effectively trained and gradually learns to make better decisions. However, the reward curve for the “15 V2V link” case exhibits larger fluctuations and a slower convergence rate compared to the other two cases. This phenomenon arises from the significantly larger action space and increased mutual interference among the numerous V2V and V2I links in this setting, which intensifies the non-stationarity and complexity of the environment, thereby making the learning process more challenging.

Table 3 presents the simulation results obtained during the process of determining the optimal value of *δ*, under the configuration of 8 V2V links, 4 V2I links, and a packet size of 6 × 1060 Bytes. As shown in the table, the best performance is achieved when *δ* = 5. The channel resource allocation task is conducted in a highly dynamic and stochastic environment. When *δ* is too large, the historical information becomes outdated due to channel variations over time, leading to ineffective state representation. Conversely, when *δ* is too small, the amount of accumulated historical data is insufficient to capture meaningful temporal patterns in channel conditions. As a result, the performance exhibits a unimodal trend-first increasing and then decreasing-as observed in Table 3. Based on this empirical evaluation, the value of *δ* is set to 5 in this study.

**Figure 6:**
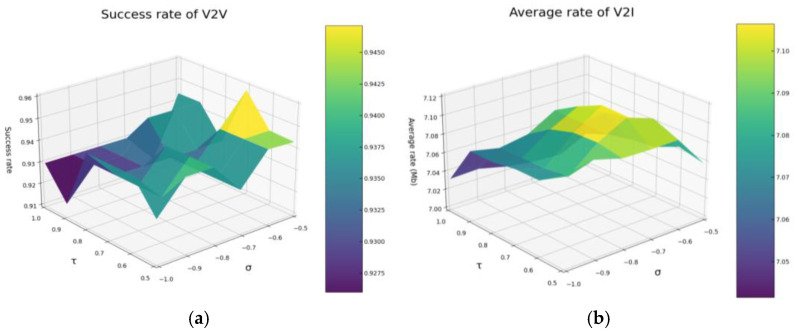
(**a**) illustrates the variation of V2V transmission success rate with respect to *σ* and *τ*. (**b**) illustrates the variation of V2I transmission rate with respect to σ and *τ*.

Figure 6 presents the simulation results for determining the optimal values of σ and *τ*, under the configuration of 8 V2V links, 4 V2I links, and a packet size of 6 × 1060 Bytes. Given that *σ* and *τ* are interrelated within the same formula, they must be discussed together. Figure 6a shows the surface plot of the V2V transmission success rate, while Figure 6b provides the surface plot of the average V2I transmission rate. Observations from Figure 5 reveal that parameter combinations yielding high V2I transmission success rates and high average V2I transmission rates are predominantly located near the diagonal. This suggests that both the V2V transmission success rate and the average V2I transmission rate are influenced not only by the absolute values of *σ* and *τ*, but also by the difference between them. Based on these findings, this paper sets *σ* to −0.7 and *τ* to 0.7. These settings aim to achieve an optimal balance between V2V transmission success and V2I average transmission rates, thereby maximizing overall system performance.

**Figure 7:**
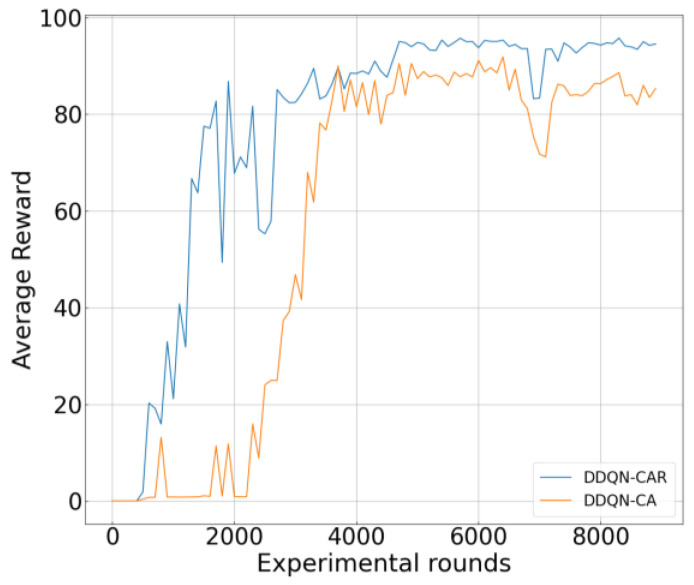
Training Curves with Different Reward Functions. DDQN-CAR employs a reward function incorporating a weighted success rate constraint $r^{\prime }=r+\lambda \cdot p^{g}$. Here, $r^{\prime }$ denotes the reward function of DDQN-CAR, and $p^{g}$ represents the global success rate, as given in Equation (10).

Figure 7 illustrates the convergence curves of the two reward functions. DDQN-CAR uses the new reward value, while DDQN-CA uses the original reward value. As shown in the figure, both reward functions achieve stable convergence, though their convergence points differ. Since DDQN-CAR includes an additional success rate gain term in its reward function, its final convergence value is higher than that of DDQN-CA. However, in subsequent performance evaluations, both methods exhibit comparable system performance. This phenomenon can be explained by the following two reasons: (a) The optimization objectives of this study encompass both maximizing transmission rate and maximizing transmission success rate. Although the reinforcement learning reward function defined in Equation (16) is presented in a weighted form, explicit constraints on the transmission success rate were imposed during the problem modeling stage. During model training, any state violating these constraints results in a significantly low rate reward, thereby being autonomously avoided by the agent. (b) Due to the difficulty in completing payload transmission within extremely short time intervals, the transmission success rate therefore exhibits minimal fluctuations across adjacent time slots. Therefore, the primary optimization driver of the reward function remains the weighted sum rate. From a communication system perspective, higher transmission rates often rely on favorable channel conditions and signal-to-noise ratios, which simultaneously provide a physical layer foundation for improving transmission success rates. Thus, continuous optimization of the weighted sum rate inherently and effectively drives the fulfillment of success rate constraints, indicating that the two objectives share an inherently consistent optimization direction at the system level. Based on this, and to maintain the simplicity of the reward function, Equation (16) is ultimately adopted as the reward function for training in this study.

**Figure 8:**
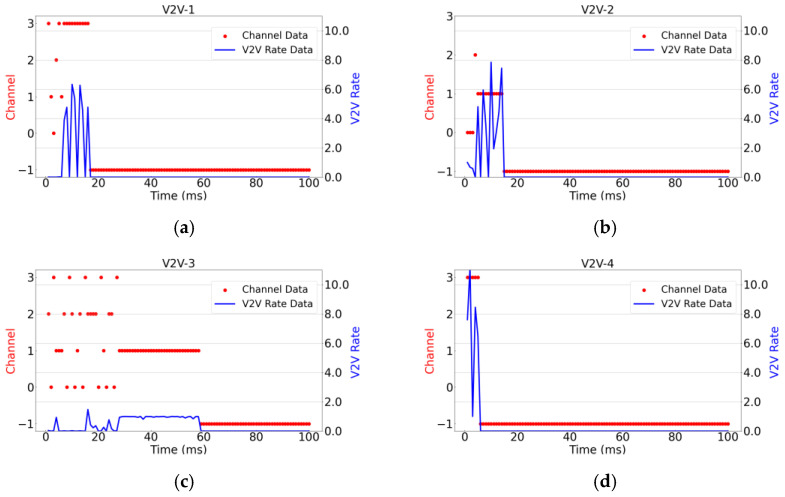

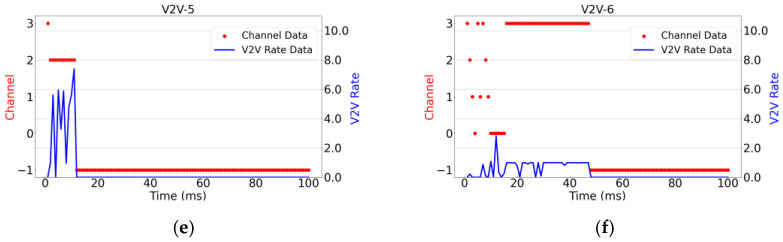
Instantaneous transmission rate and channel selection plot. (**a**–**f**) illustrate the instantaneous variations of channel selection and rate for the six V2V links, V2V-1 through V2V-6, during one transmission cycle.

Figure 8 illustrates the relationship between instantaneous transmission rates and channel selection for six randomly selected V2V links over one period, under the configuration of 4 V2I links, 8 V2V links, and a packet size of 4 × 1060 Bytes for V2V links. The figure depicts the channel selection and corresponding transmission rates of each V2V link within a transmission period as they vary over time. Since this set of simulation experiments aims to reflect the instantaneous changes in channel conditions and transmission rates over time, no averaging has been performed. In the figure, −1 indicates that the channel is off, while the values 0, 1, 2, and 3 on the left vertical axis represent the four channels used in this experiment. The right vertical axis indicates the transmission rate values of the V2V links. This visualization provides insights into how dynamically changing channel conditions affect the transmission performance of individual V2V links within a given period, without smoothing out short-term variations through averaging.

As shown in Figure 8, this experiment tracks two key metrics for each agent over time: the selected communication channel and the achieved transmission rate. By observing Figure 8c,e, it can be readily observed that once these agents find a channel capable of satisfying their transmission requirements, they do not continue to compete for potentially better channel resources to achieve higher rates. Furthermore, although the maximum achievable rate for V2V links exceeds 10, none of the agents persistently contend for optimal channels once their transmission needs are met. Instead, after securing a suitable channel, each agent refrains from further competition. This behavior demonstrates that the proposed algorithm enables effective cooperation among multiple agents. Moreover, Figure 8 shows that the V2V-1, V2V-2, V2V-4, and V2V-7 links complete their transmission tasks first and subsequently deactivate their communication links. Following this, V2V-3 and V2V-5 quickly perceive the change in the environment—specifically the reduction in channel contention—and promptly switch to channel 1 and channel 2, respectively, for their transmissions. This dynamic response clearly illustrates that the proposed algorithm is capable of sensitively detecting environmental changes and reacting swiftly to them. Additionally, by examining the transmission rates during the initial 20 ms across all subplots in Figure 8, it is evident that not every V2V link successfully finds a suitable channel within this period. This is due to the limited number of available channels—only one-third of the total number of transmission links—which results in intense competition. Differences in individual link conditions and experienced interference further contribute to varying access delays. In summary, through comprehensive analysis of Figure 8, it is demonstrated that the proposed algorithm enables agents to rapidly adapt to dynamic environments and exhibit cooperative behavior, effectively balancing resource utilization and system stability under constrained spectrum conditions.

**Figure 9:**
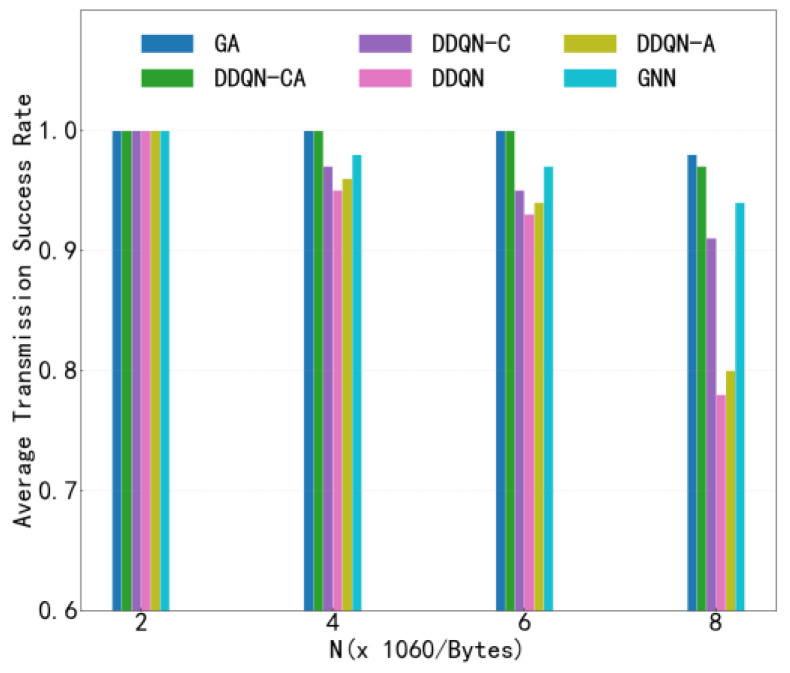
Transmission Success Rate.

Figure 9 illustrates the variation in V2V transmission success rate with respect to the number of transmitted payloads under the configuration of 4 V2V links, comparing several different algorithms. This figure evaluates the performance of each algorithm in terms of V2V transmission reliability. As shown in the figure, the success rates of all algorithms decrease as the number of transmitted payloads increases. This degradation is primarily attributed to the longer transmission duration required for larger payloads, which intensifies the temporal overlap and mutual interference among V2V links. Notably, the proposed algorithm consistently achieves a higher V2V transmission success rate compared to baseline methods when transmitting the same number of payloads, demonstrating its superior efficiency. This improvement stems from the enhanced cooperation among agents enabled by the proposed approach, which effectively reduces inter-agent interference and promotes coordinated channel access. Under high payload loads, the proposed method performs slightly worse than the centralized heuristic algorithm; however, it still outperforms all other distributed or learning-based baselines. The marginal gap with the centralized heuristic highlights a favorable trade-off between performance and scalability, as the proposed method operates in a fully distributed manner without requiring global coordination.

**Figure 10:**
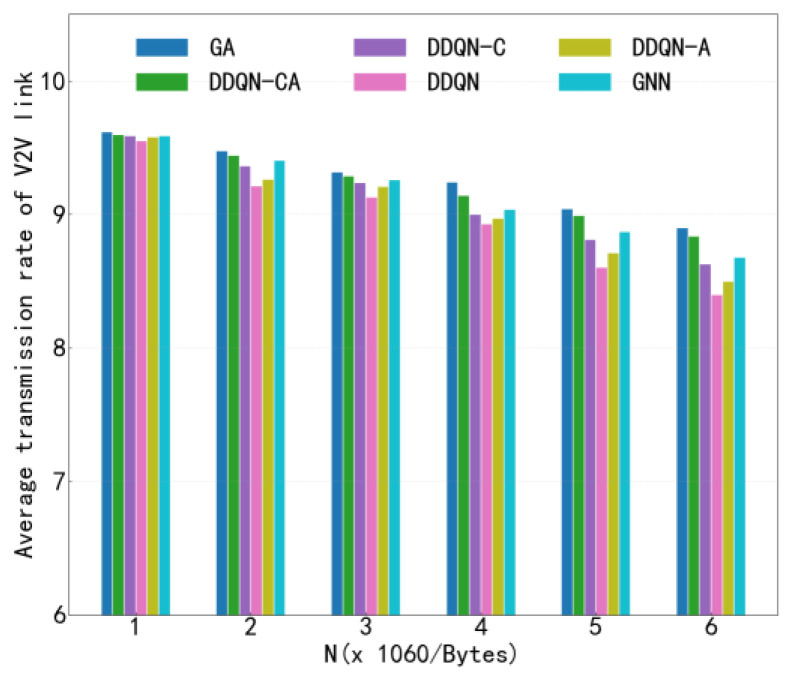
Average Transmission Rate.

Figure 10 presents the average V2I transmission rate of different algorithms under the configuration of 4 V2V links, as the number of V2V transmitted payloads increases. This figure evaluates the impact of V2V traffic load on the performance of V2I communications. As observed in Figure 10, the average V2I transmission rate decreases for all algorithms as the V2V payload load increases. This degradation is caused by the prolonged transmission duration and increased channel occupancy of V2V links, which in turn leads to more frequent and sustained interference on V2I communications. Notably, the proposed algorithm achieves a comparable or higher average V2I transmission rate than the baseline methods under the same payload load, demonstrating its ability to better mitigate cross-link interference. By enabling intelligent and cooperative spectrum access among agents, the proposed approach effectively preserves V2I communication quality even under high V2V traffic loads. When comparing Figure 9 and Figure 10, it is evident that, under identical payload conditions, the proposed algorithm outperforms existing methods in both V2V transmission success rate and average V2I transmission rate. This simultaneous improvement in both metrics highlights the effectiveness of the proposed method in achieving a favorable balance between V2V reliability and V2I throughput, thereby enhancing the overall performance of the C-V2X network.

**Figure 11:**
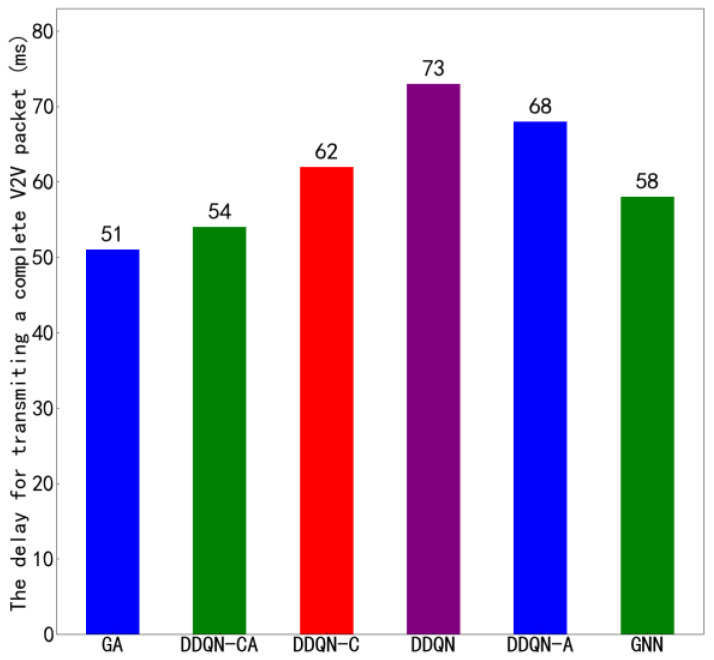
Average Transmission Delay of 4 V2V Links.

**Figure 12:**
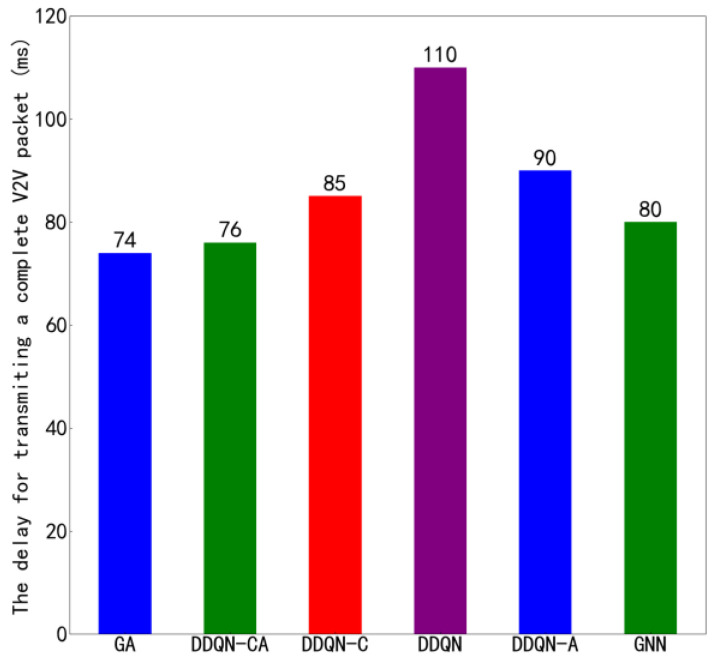
Average Transmission Delay of 8 V2V Links.

Figure 11 shows the average transmission time of the proposed algorithm compared to other algorithms under the configuration of 4 V2V links and 4 V2I links, with a total of 6 data packets being transmitted. Figure 12 presents similar results but under the configuration of 8 V2V links and 4 V2I links, also transmitting 6 data packets. By observing Figure 10 and Figure 12, it can be seen that the proposed algorithm achieves lower average transmission times than the other algorithms in both configurations. Specifically, under the settings with 4 V2V links and 8 V2V links, the proposed method consistently outperforms its counterparts, indicating its superior efficiency in managing transmission delays. These results highlight the effectiveness of the proposed algorithm in optimizing resource allocation and reducing interference among concurrent transmissions, thereby enhancing overall system throughput and reliability.

The suboptimal data transmission performance of V2V links is primarily attributable not to the data transmission process itself, but to packet collisions. In conventional distributed algorithms, each link independently selects the optimal channel, which frequently leads to multiple links simultaneously contending for the same high-quality resources, thereby inducing severe interference and packet collisions. As observed in Figure 8, Figure 9, Figure 10, Figure 11 and Figure 12, the proposed algorithm in this work, through collaborative learning among agents, enables the links to distribute themselves more evenly across different channel resources. This results in more balanced channel occupancy, effectively avoiding collisions with other links and allowing each link to select resource combinations that minimize interference. Consequently, as demonstrated in Figure 9 and Figure 10, the proposed algorithm achieves significantly superior performance in the key metric of average transmission time compared to other benchmark algorithms, fully validating the effectiveness of its design in reducing end-to-end delay.

**Figure 13:**
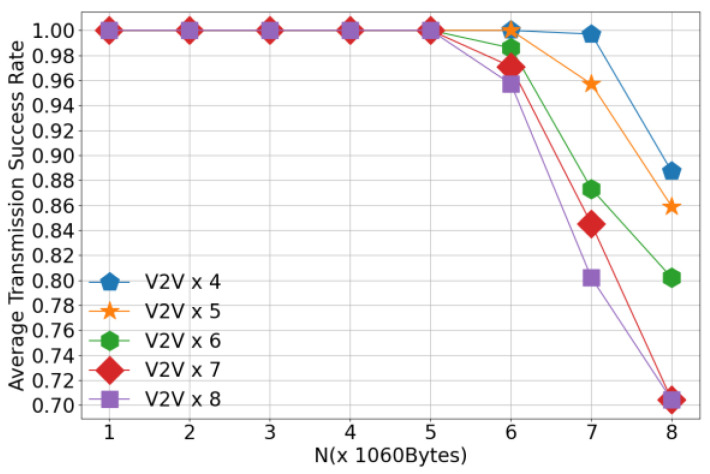
V2V Transmission Success Rate versus Payload Load.

Figure 13 illustrates the V2V transmission success rate as a function of payload load under different numbers of V2V links. As shown in the figure, when the payload load is fixed, the V2V transmission success rate decreases with an increasing number of V2V links. This degradation is primarily caused by the heightened interference among V2V links due to greater channel contention in denser network scenarios. Furthermore, for a fixed number of V2V links, the success rate declines as the payload size increases. This is because larger payloads require longer transmission durations, which extend the time intervals during which V2V links interfere with each other. The prolonged channel occupancy intensifies mutual interference, thereby reducing the probability of successful packet delivery. These results highlight the challenges of reliable V2V communication under high traffic density and large data demands.

**Figure 14:**
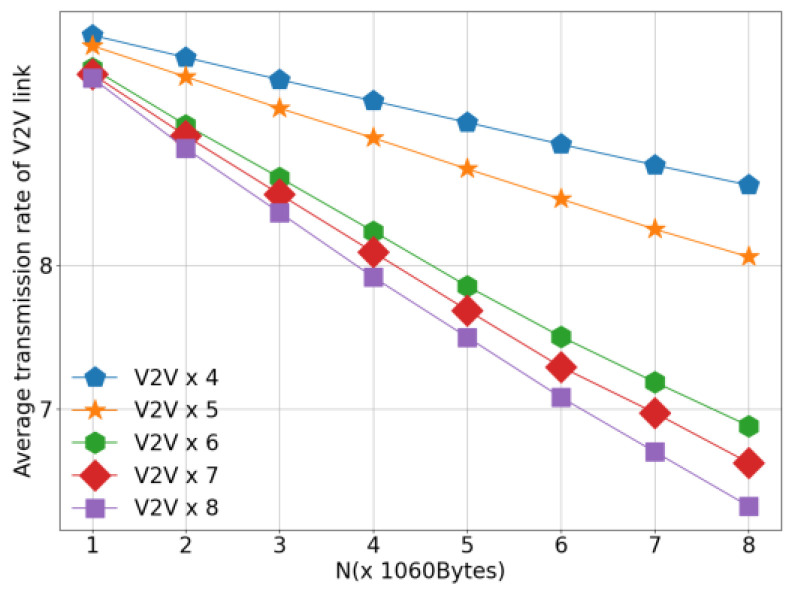
V2I Transmission Rate versus Payload Load.

Figure 14 illustrates the variation in the average V2I transmission rate with respect to the number of V2V transmitted payloads under different numbers of V2V links. The figure provides insights into how the presence of additional V2V links and increased payload sizes impact V2I communication performance. As observed in Figure 14, for a fixed payload size, the average V2I transmission rate decreases as the number of V2V links increases. This decline is attributed to the increased competition for channel resources between V2V and V2I links. More V2V links lead to higher levels of noise and interference, which adversely affect the reliability and throughput of V2I communications. Additionally, for a fixed number of V2V links, the average V2I transmission rate also decreases as the payload size increases. This reduction occurs because larger payloads require longer transmission times for V2V links, thereby extending the periods during which V2I links experience interference from V2V transmissions. The extended interference duration negatively impacts the overall performance of V2I links. These observations underscore the significant influence of V2V traffic on V2I communication quality, particularly in terms of channel contention and interference. Despite these challenges, the proposed algorithm demonstrates its effectiveness by mitigating the adverse effects of increased V2V activity and maintaining robust V2I performance under varying network conditions. This highlights the algorithm’s capability to balance the competing demands of V2V and V2I communications in dynamic vehicular environments.

The declining trend in the transmission performance of both V2V and V2I links with increasing payload volume, as shown in Figure 9, Figure 10, Figure 13 and Figure 14, can be understood from the following two perspectives. From the collision perspective, the increase in payload directly leads to a higher frequency of transmission attempts and an extended duration per transmission within the network. This significantly raises the probability of multiple links transmitting data concurrently over the same time-frequency resources, resulting in a nonlinear increase in signal collision events. For V2V links, collisions directly cause packet loss, leading to a decline in transmission success rate. For V2I links, interference induced by collisions occupies available bandwidth, thereby reducing the average transmission rate. From the channel occupancy perspective, the larger data volume substantially increases the proportion of time the channel remains in a busy state. The sharp reduction in available transmission windows intensifies competition among all links for limited resources. As V2V links struggle to secure transmission opportunities within the required low-latency constraints, their success rate decreases accordingly. Meanwhile, for V2I links, which typically rely on base station coordination, the allocable continuous or stable bandwidth resources are compressed.

**Figure 15:**
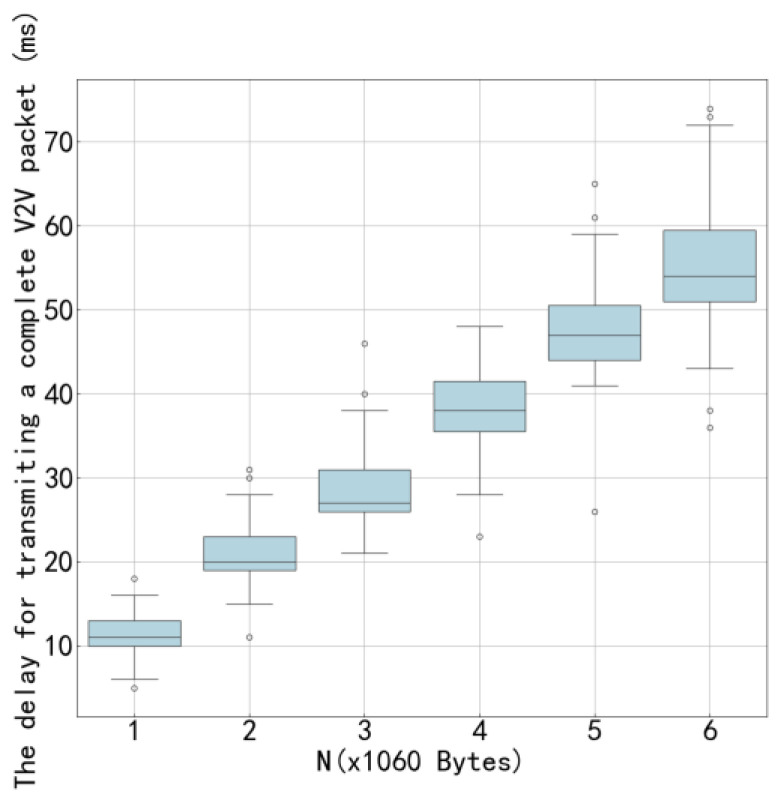
Transmission Delay as a Function of Load for 4 V2V Links.

**Figure 16:**
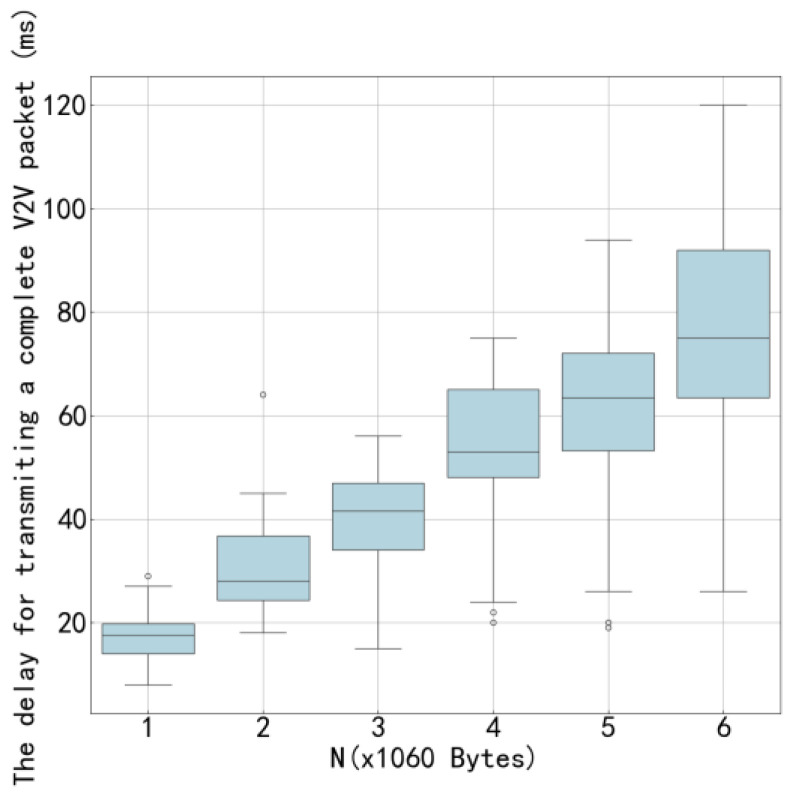
Transmission Delay as a Function of Load for 8 V2V Links.

Figure 15 and Figure 16 illustrate the variation in V2V transmission delay with respect to payload load under two different configurations: 4 V2V links and 8 V2V links, respectively. As observed in both figures, the V2V transmission delay increases as the payload size grows, which is expected due to longer transmission durations required for larger data volumes. Moreover, by comparing Figure 15 and Figure 16, it can be seen that, under the same payload load, the transmission delay in the 4-V2V-link scenario is significantly lower than that in the 8-V2V-link scenario. This performance gap arises because, under fixed communication resources, a higher number of V2V links leads to increased channel contention and interference, resulting in more frequent access delays and retransmissions. The increasing trend in transmission delay with payload load is primarily attributed to the finite system capacity. When the total transmission workload increases-either through larger payloads or a greater number of active links-the time required to complete all transmissions increases accordingly. In dense scenarios with 8 V2V links, this effect is further exacerbated by intensified mutual interference and reduced per-link resource availability. The proposed algorithm demonstrates effective delay control across both network scales, particularly by enabling cooperative spectrum access and reducing redundant collisions, thereby improving overall system efficiency even under constrained resources.

## 5. Discussion and Conclusions

Current research on communication resource allocation algorithms in vehicular networks primarily focuses on mitigating interference among different links, enhancing spectrum efficiency, and addressing the challenges posed by dynamically increasing vehicle densities. The state-aware resource allocation algorithm proposed in this paper improves the transmission success rate of V2V links and the average throughput of V2I links by enhancing collaboration among intelligent agents. However, the performance of the proposed algorithm degrades as vehicle density increases-a phenomenon commonly observed across existing studies in this field. The author has conducted an in-depth investigation into the root cause of this performance degradation and plans to further explore how to ensure stable performance of the resource allocation algorithm in scenarios with dynamically changing vehicle density in subsequent work. The author will also focus on maintaining the stability of the algorithm, the transmission success rate of V2V links, and the average transmission rate of V2I links in scenarios with increasing vehicle density.

## Figures and Tables

**Table 3:** Performance of the algorithm under different values of *δ*.

*δ*	3	4	5	6	7
Success rate of V2V	0.916	0.929	0.957	0.952	0.937
Average rate of V2I	7.066	7.069	7.079	7.052	7.037

## Data Availability

The original contributions presented in this study are included in the article. Further inquiries can be directed to the corresponding author.
